# Synergistic effects of action observation and object affordance on cortical excitability during BCI-triggered motor imagery in stroke: a randomized, within-subject, 2 × 2 factorial study protocol

**DOI:** 10.3389/fnhum.2026.1883926

**Published:** 2026-09-14

**Authors:** Yajuan Su, Xirui Yan, Weiying Li, Yixin Wang, Xubo Wu

**Affiliations:** 1 Department of Rehabilitation Medicine, Shanghai Pudong New Area People’s Hospital, Shanghai, China; 2 College of Rehabilitation Medicine, Jiamusi University (The Third Affiliated Hospital of Jiamusi University), Jiamusi, China; 3 School of Rehabilitation Science, Shanghai University of Traditional Chinese Medicine, Shanghai, China

**Keywords:** action observation, brain-computer interface, event-related desynchronization, motor imagery, object affordance, stroke rehabilitation

## Abstract

**Background:**

Brain-computer interfaces (BCI) using motor imagery (MI) offer promising rehabilitation potential for upper limb function in stroke survivors. Conventional paradigms rely on abstract visual feedback, which may lack ecological validity and could limit neural recruitment. Action observation (AO) and object affordance (OA) independently modulate motor cortical excitability via the mirror neuron system and canonical neural pathways. However, their potential synergistic effects in enhancing BCI performance have not been systematically investigated. We propose a novel “Phygital” intervention integrating dynamic visual cues (AO) with static environmental cues (OA) to potentially enhance cortical excitability and support motor rehabilitation.

**Methods:**

We employed a randomized within-subject 2 × 2 factorial design to disentangle dynamic and static visuomotor components during grasping MI. Participants include subacute stroke survivors with hemiplegia and age-matched healthy controls. Four conditions will be tested: (A) Baseline (pureMI), (B) AO (video-guided imagery), (C) OA (object-focused imagery), and (D) Synergy (simultaneous AO and OA). A 32-channel electroencephalography quantified event-related desynchronization (ERD) in mu and beta bands over sensorimotor cortex. Surface electromyography monitored muscle quiescence. Significant task-related ERD triggered a soft robotic hand exoskeleton to deliver time-locked proprioceptive feedback, closing the sensorimotor loop.

**Hypothesis:**

We hypothesize that congruent simultaneous presentation of AO and OA cues will produce a significant AO × OA interaction in sensorimotor ERD. Specifically, the facilitatory effect of AO on ERD is expected to be greater when OA is present than when OA is absent. This interaction is also expected to be accompanied by improved BCI performance compared with the single-cue conditions.

**Significance:**

By anchoring MI to tangible physical targets within a digital sensorimotor loop, this protocol bridges covert motor planning and real-world interaction. Findings will elucidate visuomotor integration mechanisms and inform development of ecologically valid, high-efficacy BCI rehabilitation strategies.

**Clinical trial registration:**

https://www.chictr.org.cn/showproj.html?proj=292325, Chinese Clinical Trial Registry, ChiCTR2500111192, prospectively registered on October 27, 2025.

## 1 Introduction

Stroke, an acute cerebrovascular event resulting in focal neurological deficits, remains a major global health challenge (Hilkens et al., 2024). It is characterized by high rates of incidence, recurrence, disability, and mortality, imposing a staggering economic and social burden on healthcare systems worldwide (Hilkens et al., 2024). Among stroke survivors, 33–80% experience persistent upper limb hemiparesis (Heller et al., 1987; Langhorne et al., 2009; Hirayama et al., 2023), with the recovery of hand function being particularly challenging due to its complex cortical representation (Buma et al., 2013; Raghavan, 2015; Hirayama et al., 2023). Recovery is particularly difficult for patients with flaccid paralysis, where the deficit in voluntary motor control significantly hinders engagement in traditional active training (Ito et al., 2025). Brain-Computer Interfaces (BCI) have emerged as a promising solution to bridge this gap by detecting Motor Imagery (MI) signals to control external devices, thereby closing the sensorimotor loop and inducing Hebbian plasticity (Mrachacz-Kersting et al., 2016; Remsik et al., 2016; Ma et al., 2025). However, current BCI paradigms suffer from limited ecological validity, as they predominantly rely on abstract, screen-based feedback disconnected from tangible environmental contexts (Ma et al., 2024; He et al., 2025; Lu et al., 2025). In reality, human motor control is not an isolated internal command but a dynamic interaction with the physical world. This discrepancy between abstract MI and physical object interaction may lead to suboptimal neural recruitment, thereby hindering the translation of BCI proficiency to functional daily activities.

To enhance the biological relevance of BCI training, we must leverage the brain’s innate capacity for motor simulation through the Action Observation Network (AON) (Sale and Franceschini, 2012; Wang and Jiang, 2023). The discovery of the Mirror Neuron System (MNS) in the premotor and parietal cortices has significantly advanced our understanding of motor cognition (Small et al., 2012; Cook et al., 2014; Kemmerer, 2021). These neurons discharge both when an individual executes a specific motor act and when they observe the same act performed by another (Brihmat et al., 2018). Capitalizing on this mechanism, Action Observation Therapy (AOT) in stroke rehabilitation utilizes the visual observation of transitive actions, such as hand grasping, to recruit the observer’s motor system in a somatotopic manner, thereby inducing “motor resonance” (Sale and Franceschini, 2012). By integrating dynamic visual cues of human action into BCI paradigms, we can potentially lower the threshold for motor cortex activation and provide a kinematic template that abstract feedback may not adequately provide.

Parallel to the mirror system, the motor cortex is also primed by the physical properties of objects in our environment, a concept known as Object Affordance (Mecklinger et al., 2004). Neurophysiological investigations have demonstrated that “canonical neurons” in the ventral premotor cortex (area F5) encode object affordances, showing activation upon the presentation of graspable objects independently of motor execution (Maranesi et al., 2019). Such activity represents a visuomotor transformation mechanism relying on the dorsal visual stream, specifically the reciprocal connections between the Anterior Intraparietal Area (AIP) and the ventral premotor cortex (Taubert et al., 2010; Lanzilotto et al., 2019). Through this pathway, the presence of an object can trigger a “pragmatic description,” instantiating the appropriate motor plan for hand orientation and grip aperture (Townsend et al., 2011). Consequently, the availability of a real physical object provides essential visuospatial coordinates and object-specific features that facilitate motor planning. This sensory component is frequently overlooked in standard, screen-only BCI protocols.

Whereas Action Observation (AO) dictates the kinematic aspects of execution, Object Affordance (OA) defines the target-related properties; in ecological contexts, these two processes function synergistically to facilitate fluid movement. We propose the Synergy Hypothesis, which suggests that the effect of dynamic AO cues on cortical excitability may be enhanced in the presence of static OA cues, resulting in an interaction between AO and OA. We posit that seeing the object “primes” the canonical neurons, thereby lowering the activation threshold for the mirror neuron system triggered by the action video. To test this, this study employs a randomized, within-subject, 2 × 2 factorial design to systematically dismantle the sensory components of a grasping task. Through the quantification of independent and interactive contributions of AO and OA to BCI accuracy and cortical oscillatory dynamics (specifically ERD), we aim to characterize a novel “Phygital” intervention model. This approach is designed to functionally integrate precise neural decoding with clinically relevant motor rehabilitation outcomes.

## 2 Objectives and hypotheses

### 2.1 Primary objective

To systematically quantify the independent and interactive effects of AO (dynamic visual cues) and OA (static environmental cues) on cortical excitability during BCI-based MI in stroke survivors and healthy controls, and to determine whether these effects differ between the two groups.

### 2.2 Secondary objectives

1. *Cortical dynamics*: To compare the spatiotemporal patterns of Event-Related Desynchronization (ERD) in Mu and Beta rhythms across four experimental conditions.
2. *Motor preparation*: To analyze the Movement-Related Cortical Potential (MRCP), specifically the amplitude and latency of the Bereitschaftspotential (Readiness Potential), to determine if ecological cues enhance early motor planning.
3. *Clinical correlation*: To explore the relationship between neurophysiological markers (ERD and MRCP magnitude) and clinical motor function and cognitive measures, including Fugl-Meyer Assessment scores (FMA-UE) and Montreal Cognitive Assessment (MoCA) scores.

### 2.3 Hypotheses

*H1*: The AO condition will elicit significantly greater ERD and MRCP amplitude compared to the Baseline (pure imagery) condition, confirming MNS activation.

*H2*: The OA condition will elicit significantly greater ERD and MRCP amplitude compared to Baseline, confirming Canonical neuron activation.

*H3*: The combined condition (AO + OA) will show a significant AO × OA interaction in sensorimotor ERD. Specifically, AO is expected to produce stronger ERD modulation in the presence of OA than in the absence of OA.

*H4*: Both groups are expected to show greater ERD during the AO and OA conditions than during Baseline MI, with the greatest ERD during the combined AO + OA condition. Compared with healthy controls, stroke participants are expected to show broader bilateral activation, while the combined condition may promote a relative shift toward the ipsilesional sensorimotor cortex.

## 3 Methods

This protocol is reported in accordance with the applicable SPIRIT 2025 recommendations (Chan et al., 2025).

### 3.1 Study design

This is a single-center, randomized-order, counterbalanced, mixed factorial experimental study. AO (absent vs. present) and OA (absent vs. present) are two within-participant factors, generating four experimental conditions. Participant group (stroke vs. healthy control) is a between-participant factor. Each participant will complete all four experimental conditions during a single experimental session. The order of the four conditions will be randomized and counterbalanced within each participant group.

### 3.2 Participants

Stroke participants will be recruited from the Department of Rehabilitation Medicine at Shanghai Pudong New Area People’s Hospital. Healthy controls will be recruited from local community and frequency-matched to the stroke group in terms of sex and mean age (±5 years). Recruitment will continue until the pre-specified sample size is reached. All participants will be stratified into two groups: the Stroke Group and the Healthy Control Group.

#### 3.2.1 Inclusion criteria

Stroke group:

(1) Meeting the *criteria of the Chinese Guidelines for the Diagnosis and Treatment of Acute Ischaemic Stroke 2023* (Chinese Society of Neurology and Chinese Stroke Society, 2024); (2) First-ever stroke with left-sided hemiplegia; (3) Right-handed; (4) Duration of illness between 1 and 6 months (subacute stage); (5) (MoCA score≥22 (Wei et al., 2023); (6) Sufficient core strength to sit steadily; (7) Age between 30 to 80 years.

Healthy control group:

(1) Right-handed; (2) No history of neurological disorders; (3) No impairment of limb motor function; (4) Normal or corrected-to-normal vision; (5) Age and gender matched with the stroke group.

#### 3.2.2 Exclusion criteria

(1) Presence of skull defects, scalp lesions, or intracranial metal implants compromising EEG safety or signal quality; (2) Severe hemispatial neglect, apraxia, or sensory aphasia preventing task comprehension or compliance; (3) Uncontrolled epilepsy, active infection, or severe cardiac, renal, or hepatic failure; (4) Use of CNS-acting medications within 24 h prior to the experiment; (5) Severe lesions involving key mirror neuron regions; (6) Participants exhibiting severe deficits in MI ability, defined as an average score of < 2 on the Kinesthetic and Visual Imagery Questionnaire (KVIQ-10), to ensure sufficient cognitive engagement for the BCI tasks (Malouin et al., 2007).

### 3.3 Apparatus and data acquisition

The experimental setup integrates three synchronized hardware systems: a high-temporal-resolution EEG acquisition unit for neural decoding, a soft hand rehabilitation robot for proprioceptive feedback, and a surface electromyography (sEMG) unit for artifact control.

#### 3.3.1 EEG acquisition

EEG signals will be acquired using a 32-channel NeuroHUB Wireless EEG System (Neuracle, Changzhou, China) with Ag/AgCl wet electrodes placed per the 10–20 system. Electrodes cover frontal, central, parietal, and temporal regions, targeting sensorimotor cortex (C3, Cz, and C4) and frontoparietal mirror neuron network. Ground (AFz) and reference (CPz) electrodes are positioned accordingly. Data will be digitized at 1000 Hz (24-bit resolution), with electrode impedances maintained <5 kΩ. Wireless transmission via dedicated WiFi eliminats 50/60 Hz interference.

#### 3.3.2 Robotic hand exoskeleton

Hand movements will be assisted by the SRH 700 Soft Hand Rehabilitation Robot (Xirun Medical, Shanghai, China), employing pneumatic artificial muscles (PAMs) for compliant, safe actuation. Dorsal finger actuators passively flex MCP/PIP joints (simulating palmar grasp) upon TTL trigger from the BCI unit upon significant ERD detection. System latency is <200 ms post-trigger to align with Hebbian plasticity time windows. The robotic hand serves as a standardized BCI-contingent proprioceptive feedback device across all four experimental conditions, thereby maintaining the closed sensorimotor loop after successful MI-related ERD detection.

#### 3.3.3 sEMG

sEMG data (1,000 Hz, 24-bit, synchronized with EEG) will be recorded from Flexor Digitorum Superficialis (FDS) and Extensor Digitorum Communis (EDC) of the affected limb using bipolar Ag/AgCl electrodes. Signals will be rectified and smoothed (RMS, 50 ms window). Trials with EMG activity >2 SD above baseline will be excluded to ensure cortical activation reflected central motor commands, not peripheral muscle activity.

### 3.4 Experimental conditions

Data collection will take place in a dedicated shielded chamber at the Rehabilitation Medicine Department of Shanghai Pudong New Area People’s Hospital. Participants will sit in an adjustable chair, 60–70 cm from a 15.6-inch eye-level monitor. The study focuses on a single functional task of grasping a medicine bottle. The experiment employs a 2 × 2 within-subject factorial design, manipulating the presence or absence of AO and OA cues. The four experimental conditions are presented in Table 1. Figure 1 schematically illustrates the four experimental conditions, showing how dynamic cues (video) and static cues (physical object) are combined during MI and influence cortical activity.

**Table 1:** The factorial matrix.

Code	AO	OA	Description	Task
A	Absent	Absent	Screen: static cross. Desk: Empty.	Imagine grasping the bottle.
B	Present	Absent	Screen: video of grasping. Desk: empty.	Watch video and imagine grasping concurrently.
C	Absent	Present	Screen: static cross. Desk: real bottle.	Look at bottle and imagine grasping.
D	Present	Present	Screen: video of grasping. Desk: real bottle.	Watch video, look at bottle, and Imagine grasping.

AO, Action Observation, A 4-s first-person perspective video showing a left hand (matching the paretic side) reaching for and grasping a medicine bottle. The video is devoid of background distractions. OA, Object Affordance, A physical medicine bottle placed on the desk within the participant’s peripersonal space (approx. 30 cm from the hand). The object is aligned with the participant’s midline to maximize the saliency of the affordance effect.

**Figure 1:**
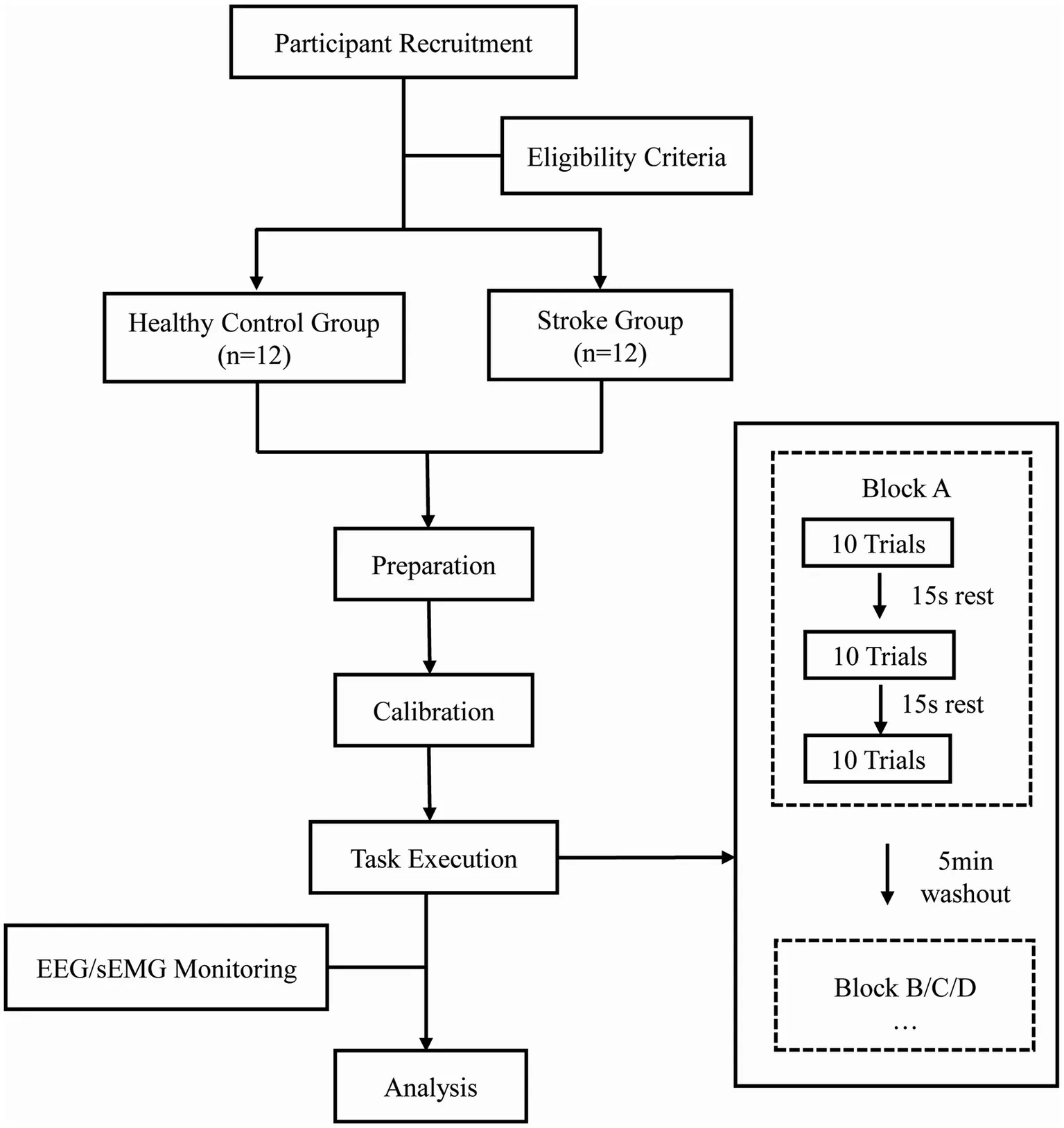
Flowchart of the experimental study design and procedural workflow.

### 3.5 Condition allocation

Given the within-subject design, randomization will apply to the order of condition presentation to mitigate sequence-dependent confounding variables, such as learning and fatigue effects. The four experimental blocks (A, B, C, D) will be counterbalanced using a 4 × 4 Latin Square design. An independent researcher will generate a computer-based allocation list. Within each participant group, participants will be randomly assigned in equal proportions to one of four Latin-square sequences: ABCD, BCDA, CDAB, or DABC. This approach ensures equal frequency of each condition across all ordinal positions, helping to minimize position biases.

The computer-based allocation list will be retained by the independent researcher and will not be accessible to the investigator responsible for participant enrollment before sequence assignment. Because the experimental conditions involve visibly different visual and object cues, blinding of participants and investigators conducting the experimental session is not feasible.

### 3.6 Experimental procedure

The schedule of enrollment, experimental procedures, and assessments is summarized in Table 2. The experimental session will last approximately 90 min, organized into three distinct phases.

**Table 2:** SPIRIT Schedule of enrollment, experimental procedures, and assessments.

Procedures	Screening/enrollment	Phase 1: preparation	Phase 2: calibration	Phase 3: main experiment	End of session
Eligibility assessment	√				
Written informed consent	√				
Clinical assessment (MoCA, KVIQ-10, FMA-UE)	√				
Condition-order allocation	√				
EEG/sEMG preparation		√			
Motor imagery familiarization		√			
BCI calibration			√		
Experimental conditions A–D				√	
EEG/sEMG recording			√	√	
5-min washout between blocks				√	
Safety/adverse-event monitoring		√	√	√	√

#### 3.6.1 Phase 1: preparation and familiarization (20 min)

Following EEG cap fitting and impedance checks, participants will undergo specific training to differentiate between Kinesthetic Motor Imagery (KMI) and Visual Motor Imagery (VMI). Participants will be explicitly instructed to use KMI (“feeling” the muscle contraction) rather than VMI (“seeing” the hand move), as KMI has been shown to elicit stronger sensorimotor cortex activation.

#### 3.6.2 Phase 2: calibration (10 min)

Participants will perform 20 trials of “Rest” versus “Motor Imagery of Grasping”. Data from this session will be analyzed offline to determine the Individual Alpha Frequency (IAF) peak and to calculate optimal Common Spatial Pattern (CSP) filter weights. To balance motivation and challenge, the classifier detection threshold will be set to maintain approximately 70% accuracy.

#### 3.6.3 Phase 3: the main experiment (45 min)

The four conditions are presented in counterbalanced blocks (30 trials/block). The trial structure includes: (1) Baseline (−3–0 s): Relaxation phase for normalization; (2) Cue (0 s): Auditory start signal; (3) Task (0–4 s): Participants perform MI while viewing the condition-specific stimulus. Successful ERD modulation triggers the robotic glove; (4) Rest (4–7 s): A jittered inter-trial interval (3–5 s) prevents rhythmic prediction. To maintain sustained attention and minimize intra-block fatigue, a short rest of 15 s is administered after every 10 trials. A 5-min washout period occurs between blocks, incorporating a cognitive distraction task (rapid serial visual counting) to mitigate fatigue and carry-over effects. A flowchart of the experimental study design and procedural workflow is shown in Figure 2.

**Figure 2:**
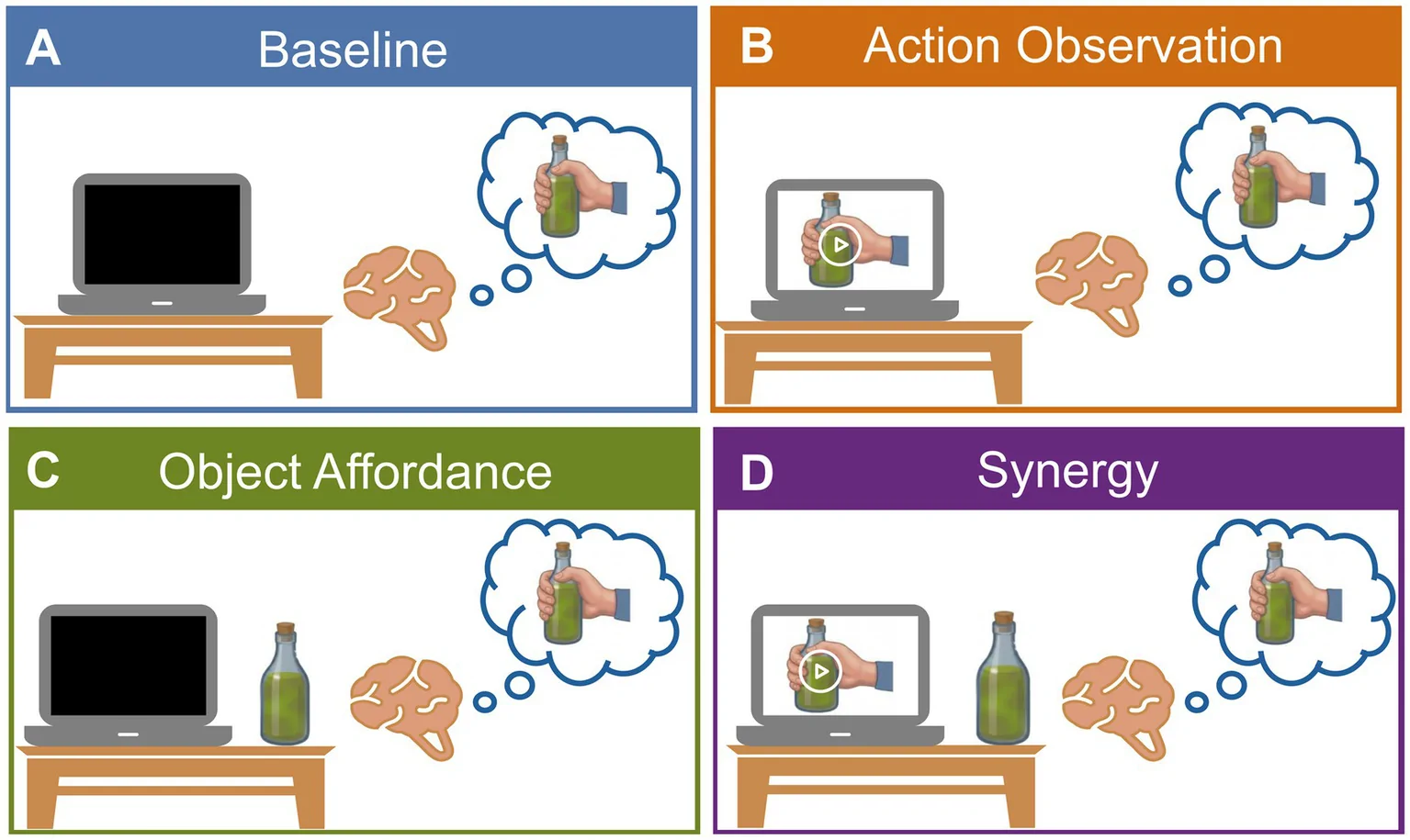
Experimental 2 × 2 factorial design. **(A)** Baseline: MI with a static screen, no object. **(B)** AO: video observation plus MI, no object. **(C)** OA: static screen with real object, MI performed with object cues. **(D)** Synergy (AO + OA): video observation and real object present, MI performed under combined cues.

#### 3.6.4 Safety monitoring

Participants will be monitored throughout the experimental session for discomfort, excessive fatigue, skin irritation related to EEG/sEMG electrodes, or discomfort associated with robotic hand assistance. Any unfavorable medical occurrence during the study procedures will be recorded as an adverse event regardless of its presumed relationship to the study. The experimental session will be paused or discontinued at the participant’s request or if clinically significant discomfort or an adverse event occurs. Any adverse event will be documented and managed by the clinical research team according to institutional procedures.

### 3.7 Outcomes

#### 3.7.1 Primary outcome: cortical activation (EEG metrics)

The primary outcome of this study is cortical activation, assessed via ERD in the sensorimotor cortex. Time-frequency analysis will be performed on 32-channel EEG signals to calculate the power spectral density (PSD) in the mu (8–13 Hz) and beta (14–30 Hz) bands relative to a baseline interval. Greater ERD (more negative values) indicates higher cortical excitability. Topographical activation maps will be generated to evaluate the spatial extent of sensorimotor engagement (C3, Cz, C4) and potential hemispheric laterality shifts, quantified using a Laterality Index, across the four experimental conditions (Baseline, AO, OA, and Synergy).

#### 3.7.2 Secondary outcome

##### 3.7.2.1 MRCP

MRCPs are slow cortical potentials observed in the motor cortex prior to voluntary movement. They reflect early motor planning and the preparatory phase before action. They are characterized by a gradual negative shift, the Bereitschaftspotential (BP), typically emerging 1–2 s prior to movement onset. Key cortical sources include the primary motor cortex (M1), supplementary motor area (SMA), and premotor cortex. MRCPs will be recorded during MI and AO tasks. Metrics of interest include BP peak amplitude and onset latency, which will quantify the effects of ecological cues on motor preparation.

#### 3.7.3 Control outcome: muscle quiescence (sEMG)

To ensure that observed cortical activations are driven by central motor commands rather than peripheral muscle activity, RMS amplitude of sEMG signals will be quantified. Trials exceeding 2 standard deviations above baseline will be excluded from EEG-based analyses. This control will help ensure that the observed EEG activity reflects MI-related cortical activity rather than overt muscle contraction.

#### 3.7.4 Technical control: online success rate

The Online Success Rate evaluates the functional reliability of the BCI system, defined as the proportion of trials in which participant-induced ERD exceeded the calibrated threshold to trigger robotic feedback. This measure complements ERD and secondary outcomes by assessing real-time system stability and functional efficacy, providing a translational readout of cortical engagement into device control.

#### 3.7.5 Data quality

EEG and sEMG data will be visually inspected and preprocessed to remove trials containing excessive noise, movement artifacts, or baseline drift. sEMG trials exceeding 2 standard deviations above baseline RMS amplitude will be excluded. EEG trials with electrode noise, drift, or muscle artifacts will be rejected using automated algorithms and visual confirmation. Only artifact-free trials will be included in EEG-based analyses to ensure that metrics reflect true cortical activity.

#### 3.7.6 Data management and confidentiality

All data files and paper records will be identified using a unique participant ID and will contain no directly identifying information. Electronic EEG, sEMG, and clinical data will be stored on a password-protected and encrypted research server, with daily incremental backups and weekly full backups. Paper documents containing identifying information, including informed consent forms, will be stored separately in locked cabinets. Two researchers will independently verify the accuracy of data entry, and data completeness will be checked regularly. Access to identifiable information will be restricted to authorized members of the research team.

### 3.8 Sample-size justification

Because reliable prior estimates specifically for the AO × OA interaction effect on ERD are currently unavailable, a total of 24 participants (12 stroke participants and 12 age- and sex-matched healthy controls) will be recruited for this exploratory mechanistic study. The study is intended to provide preliminary estimates of the magnitude and variability of the AO × OA interaction and to inform the sample-size calculation of a future confirmatory study.

### 3.9 Statistical analysis

A mixed-design repeated-measures ANOVA will be conducted for the primary ERD outcome, with AO (absent vs. present) and OA (absent vs. present) as within-participant factors and Group (stroke vs. healthy control) as a between-participant factor. The AO × OA interaction will constitute the primary statistical test of the synergy hypothesis, while the AO × OA × Group interaction will be examined to determine whether the interaction between AO and OA differs between stroke participants and healthy controls. The main effect of Group and the AO × Group and OA × Group interactions will also be examined. Significant Group-related effects will be followed by Bonferroni-adjusted between-group comparisons within each of the four conditions. Prior to the analysis, data will be tested for normality using the Shapiro–Wilk test. To test the synergy hypothesis, a prespecified interaction contrast will be applied across the four experimental conditions: (D − C) − (B − A), where A represents the Baseline condition, B represents AO alone, C represents OA alone, and D represents the combined AO + OA condition. This contrast tests whether the effect of AO differs according to the presence or absence of OA. A significant interaction in the hypothesized direction will be interpreted as evidence that OA enhances the effect of AO on ERD. Significant interactions will be followed by simple-effect comparisons, with Bonferroni adjustment for multiple comparisons.

To explore relationships between neurophysiological and clinical measures, Pearson correlation coefficients will be calculated between ERD and MRCP measures and clinical measures, including FMA-UE and MoCA scores, as appropriate. This will allow examination of how cortical excitability relates to motor function and cognitive performance. *p*-values will be corrected for multiple comparisons using Bonferroni adjustment. Effect estimates will be reported with 95% confidence intervals in addition to *p*-values. Additionally, exploratory regression analyses may be performed to investigate whether cognitive function (MoCA) predicts variability in MRCP across participants.

## 4 Discussion

The primary objective of this protocol is to evaluate the ‘Synergy Hypothesis,’ which posits that combining AO and OA may yield a synergistic facilitation of motor cortex excitability. Underpinning this hypothesis is the premise that while AO supplies the kinematic template via the Mirror Neuron System, OA recruits canonical neurons in the ventral premotor cortex (area F5) to encode pragmatic features. We anticipate that this simultaneous presentation creates a dual-stream facilitation, where the physical object primes the motor system via dorsal pathways, thereby optimizing the subsequent neural recruitment triggered by action observation.

A distinct feature of this proposed protocol is the incorporation of the Real Object Advantage. Accumulating neurophysiological evidence suggests that real, tangible objects elicit significantly more robust and sustained ERD in sensorimotor Mu and Beta rhythms compared to 2D images or virtual representations (Kumar et al., 2013; Freeman et al., 2016; de Vega et al., 2019; Xin and Zhang, 2022). These findings imply that the brain’s motor preparation networks appear to be sensitive to the actionability of the environment. By embedding physical objects into the digital BCI loop, this study aims to mitigate the limited ecological validity of conventional screen-based paradigms, potentially augmenting neural recruitment by anchoring MI to a tangible physical target.

From a methodological standpoint, the chosen 2 × 2 factorial design offers a distinct advantage by permitting direct estimation of the AO × OA interaction, thereby determining whether the effect of AO on cortical excitability depends on the presence or absence of OA (Mdege et al., 2014). Clinically, this approach may offer potential advantages for patients with severe post-stroke hemiparesis. We postulate that the combined AO-OA sensory input acts as an exogenous scaffold, compensating for compromised internal motor planning and reinforcing the sensorimotor loop necessary for BCI control. This mechanism is expected to drive Hebbian plasticity through time-locked robotic feedback. The execution of this protocol may help determine if visuomotor congruency BCI environments can effectively potentiate functional rehabilitation outcomes.

In addition to its primary focus on motor rehabilitation, this protocol may provide a basis for exploring the broader relevance of BCI-based approaches to post-stroke non-motor dysfunction, given the interdependence of motor, cognitive, and emotional recovery (Mane et al., 2020; Ma et al., 2025). The inclusion of MoCA and the analysis of ERD and MRCP may offer preliminary insight into the relationship between cognitive status and neurophysiological responsiveness during motor imagery, while the recorded beta activity may support future investigations of beta sub-band dynamics related to attention, inhibitory control, and self-regulation (Habelt, 2026). Nevertheless, dedicated longitudinal studies using validated measures of depression, anxiety, fatigue, and other non-motor symptoms are needed to evaluate these potential effects directly.

This study has several limitations. First, the sample size is modest (*n* = 24), and the study is conducted at a single center, which may limit generalizability. Given its exploratory nature, the study is primarily intended to provide preliminary estimates of the AO × OA interaction and its variability, which may inform the design and sample-size calculation of future confirmatory studies. Second, only subacute stroke patients with mild to moderate hemiparesis are included; findings may not extend to acute or severely impaired populations. Third, the study focuses on a single grasping task, which may limit applicability to other motor functions. Future studies could expand to multi-center trials, larger and more diverse cohorts, and additional functional tasks to assess broader clinical applicability and robustness of the synergistic AO + OA intervention.
